# Data for the co-expression and purification of human recombinant CaMKK2 in complex with calmodulin in *Escherichia coli*

**DOI:** 10.1016/j.dib.2016.06.033

**Published:** 2016-06-29

**Authors:** Lisa Gerner, Steffi Munack, Koen Temmerman, Ann-Marie Lawrence-Dörner, Hüseyin Besir, Matthias Wilmanns, Jan Kristian Jensen, Bernd Thiede, Ian G. Mills, Jens Preben Morth

**Affiliations:** a Centre for Molecular Medicine Norway (NCMM), Nordic EMBL Partnership, Forskningsparken, University of Oslo and Oslo University Hospitals, 0349 Oslo, Norway; b European Molecular Biology Laboratory Hamburg, Notkestrasse 85, 22603 Hamburg, Germany; c European Molecular Biology Laboratory Heidelberg, Meyerhofstrasse 1, 69117 Heidelberg, Germany; d Institute of Biochemistry, WWU Münster, Wilhelm-Klemm-Straße 2, 48149 Münster, Germany; e Department of Molecular Biology and Genetics, Aarhus University, 8000 Aarhus, Denmark; f Department of Biosciences, University of Oslo, Norway; g Biotechnology Centre of Oslo, University of Oslo, Norway; h Department of Molecular Oncology, Institute for Cancer Research, Oslo University Hospitals, Norway; i Movember/Prostate Cancer UK Centre of Excellence for Prostate Cancer Research, Centre for Cancer Research and Cell Biology (CCRCB), Queen׳s University Belfast, UK; j Institute for Experimental Medical Research, Oslo University Hospital, Norway

**Keywords:** aa, amino acid, AMPK, adenosine monophosphate-regulated kinase, CaM, calmodulin, CaMK, calcium/calmodulin-dependent kinase, CaMKK2, calcium/calmodulin-dependent kinase kinase 2, CaMKK2:CaM, CaMKK2-CaM complex, *E. coli*, *Escherichia coli*, IPTG, β-D-1-thiogalactopyranoside, LB, Luria broth, LDS-PAGE, lithium dodecyl sulphate–polyacrylamide gel electrophoresis, LIC, ligation-independent cloning, OD, optical density, PMSF, phenylmethylsulfonyl fluoride, SEC, size-exclusion chromatography, TEV, tobacco etch virus, CaMKK2, Calmodulin, Fermentation

## Abstract

Calcium/calmodulin-dependent kinase kinase 2 (CaMKK2) has been implicated in a range of conditions and pathologies from prostate to hepatic cancer. Here, we describe the expression in *Escherichia coli* and the purification protocol for the following constructs: full-length CaMKK2 in complex with CaM, CaMKK2 ‘apo’, CaMKK2 (165-501) in complex with CaM, and the CaMKK2 F267G mutant. The protocols described have been optimized for maximum yield and purity with minimal purification steps required and the proteins subsequently used to develop a fluorescence-based assay for drug binding to the kinase, “Using the fluorescent properties of STO-609 as a tool to assist structure-function analyses of recombinant CaMKK2” [1].

**Specifications Table**

**Table:** 

Subject area	Biochemistry, Molecular Biology
More specific subject area	Protein expression and purification
Type of data	Figures, table
How data was acquired	Cloning and recombinant expression technology, affinity and size exclusion chromatography
Data format	Raw, analyzed
Experimental factors	Common laboratory practises, when handling recombinant bacteria
Experimental features	*E. coli* protein expression, protein purification via affinity (NiNTA) chromatography and size exclusion chromatography
Data source location	Oslo, Norway; Heidelberg, Germany
Data accessibility	Data is with this article

**Value of the data**

• CaMKK2 has relevance as an important cancer drug target and its structural-functional relationship is not fully understood yet which makes further characterization dependent on highly pure kinase protein.
• A detailed process for expression and purification of CaMKK2 in complex with calmodulin is described.
• *E. coli* expression with subsequent affinity and size exclusion chromatography ensure proper protein purity and absence of protein aggregates.
• The data include the protocols for full-length CaMKK2, a shorter construct encompassing kinase domain and CaM-binding domain only, as well as a full-length CaMKK2 construct with a single mutation in the ATP-binding pocket.
• The methods and data described provide an experimental guide to further define structural and functional aspects of CaMKK2.

## 1 Data

We describe the cloning strategy for subsequent expression and purification of the complex of CaMKK2 and CaM, and in addition for CaMKK2 ‘apo’, a truncated construct including only residues 165 to 501 CaMKK2(165-501) in complex with CaM, and a single mutation construct CaMKK2(F267G); all primers and expression vectors are listed. Protein expression in *E. coli* Rosetta™ II (DE3) and the purification steps (affinity and size exclusion chromatography), including cleavage of the His-tag, are all validated by showing corresponding chromatograms and LDS-PAGEs.

The essential information used to generate these data are provided below in the form of expression and purification protocols.

## 2 Experimental design, materials and methods

Unless otherwise specified, all chemicals and reagents were purchased from Sigma-Aldrich in at BioUltra reagent grade.

### 2.1 Cloning strategy

**Fig. 1:**
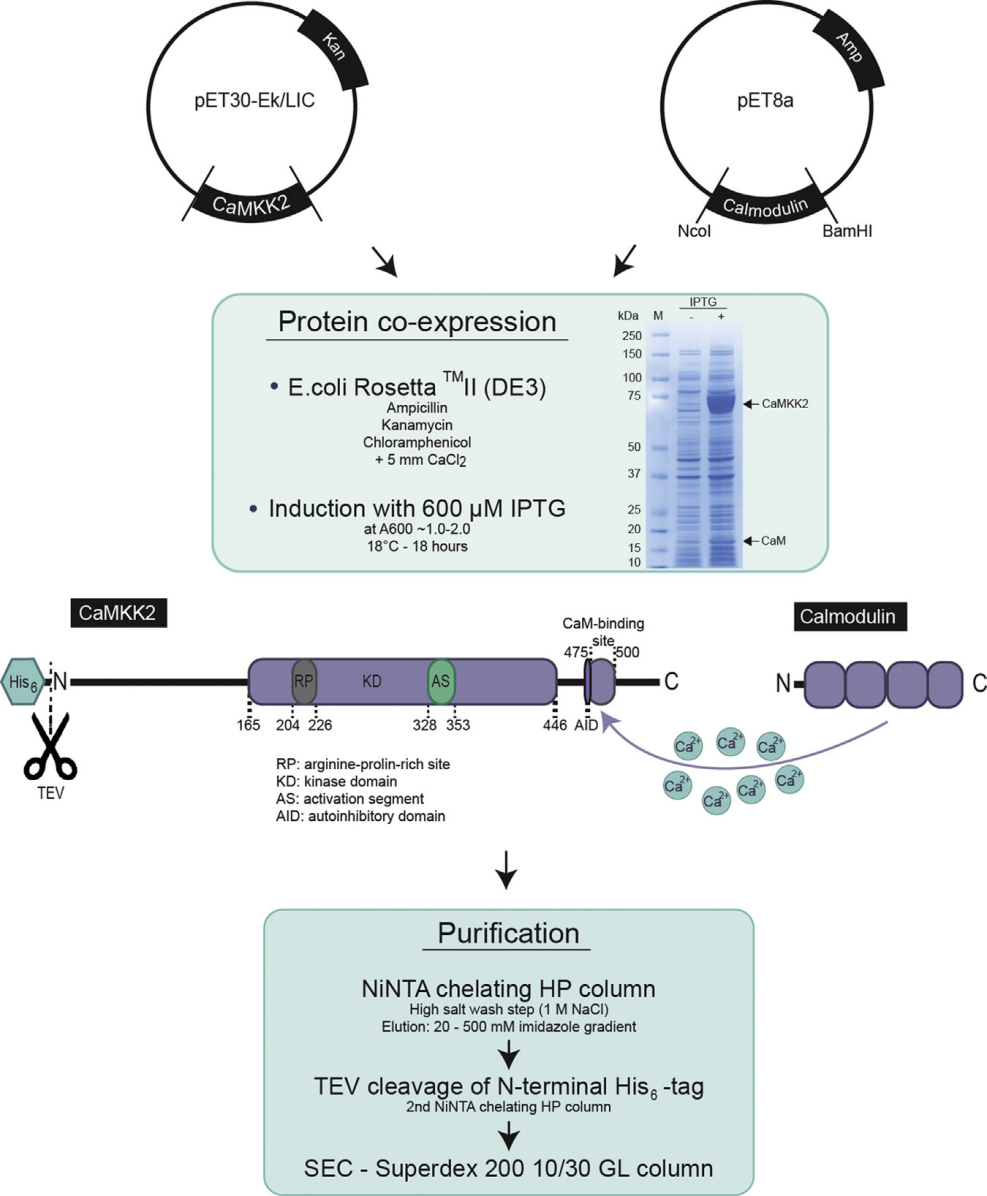
Workflow diagram. CaMKK2-pET30-Ek/LIC and CaM-pET8a were co-transformed into *E. coli* Rosetta™ II (DE3) and overexpression performed as indicated. LDS-PAGE shows samples taken before induction and 18 hours after induction; M=molecular weight marker. Bands corresponding to overexpressed CaMKK2 and CaM proteins are marked. The His_6_-tagged full-length CaMKK2 protein (541 aa) with its kinase domain (KD) (aa 165-446), arginine-proline-rich site (RP) (aa 204-226), autoinhibitory domain (AID) (aa 472-477), CaM binding domain (aa 475-500). The binding of CaM to CaMKK2 is Ca^2+^-dependent and consequently, CaCl_2_ was included throughout expression and purification. Protein purification was achieved as outlined. Affinity chromatography was followed by TEV-cleavage and dialysis. After a second affinity chromatography step the CaMKK2:CaM complex was subjected to size exclusion chromatography.

Previously, de Diego et al. [2] reported a method to co-purify CaM with a fragment of the CaM-binding kinase, DAPK1 (aa 1-334). This involved the co-transformation and co-expression of His-tagged kinase with un-tagged calmodulin (CaM). In this data, we adopted an analogous approach (Fig. 1) and used His-tagged full-length CaMKK2 and un-tagged CaM.

Therefore, the gene sequence of the full-length CaMKK2 construct was amplified by polymerase chain reaction (PCR) from a pCMV-Ac-GFP vector containing human CaMKK2 isoform 3 **(Q96RR4-3, KKCC2_HUMAN)** (Origene). The amplified gene fragment was cloned into a pET30-Ek/LIC vector (Novagen), hereafter be referred to as CaMKK2, using ligation-independent cloning (LIC) according to the manufacturer׳s instructions. With the pET30-Ek/LIC we incorporated an additional Tobacco Etch Virus (TEV) protease cleavage site in the forward primer (Table 1). Truncated constructs had also been cloned into pET28a using *Bam*HI and *Xho*I restriction sites. The truncated CaMKK2 construct containing residues 165 to 501 (CaMKK2 (165-501)) was selected to encompass the kinase domain (aa 165-446) including the putative CaM-binding site (aa 475-500). The second construct, CaMKK2 with a single mutation in the so-called ‘gatekeeper’ residue (F267G) of the nucleotide binding pocket, has been mutated using QuikChange II XL Site-Directed Mutagenesis Kit (Agilent Technologies) as to manufacturer׳s description using the CaMKK2-pET28a construct as a template. Human CaM (P62158, CALM_HUMAN) cloned into pET8a with *Nco* I and *Bam* HI sites has been kindly provided by Inaki de Diego (EMBL Hamburg). All resulting plasmids were controlled by DNA sequencing using the commercial service GATC Biotech (Konstanz, Germany). All primers used in this study (Sigma-Aldrich and GATC Biotech) are listed in Table 1.

### 2.2 Expression and purification of CaMKK2:CaM

For co-expression of the CaMKK2:CaM complex, the *E. coli* Rosetta™ II (DE3) strain was used. This strain is designed to enhance the expression of eukaryotic proteins, with reading frames that contain codons rarely used by *E. coli*. The CaMKK2 reading frame has a high number of rare codons, particularly the codon for proline (CCC) within the N-terminal coding region of CaMKK2. The *E. coli* Rosetta™ II (DE3) strain supplies tRNAs for seven rare codons on a compatible chloramphenicol-resistant plasmid [3]. We co-transformed *E. coli* Rosetta™ II (DE3) with CaMKK2-pET30-Ek/LIC and CaM-pET8a constructs by heat-shock and plated them onto agar plates. The co-transformed bacteria grow under triple antibiotic selection (ampicillin 100 μg/ml, kanamycin 25 μg/ml and chloramphenicol 35 μg/ml) since the pET30-Ek/LIC vector contains an antibiotic resistance cassette against kanamycin, and the CaM vector (pET8a) against ampicillin, and the plasmid with rare codons on *E. coli* Rosetta™ II (DE3) confer chloramphenicol resistance. A 100 ml overnight culture was used to inoculate 10 l of Luria broth (LB) medium (1% tryptone, 0.5% yeast extract (Oxoid), 0.5 % NaCl (VWR)), supplemented with triple antibiotic selection, 5 mM CaCl_2_ (included throughout expression to stabilise the CaMKK2:CaM complex) and 0.06% PEG 2000. The culture was left to grow at 37 °C using a LEX™-48 bioreactor (Harbinger Biotechnology and Engineering) to an optical density (OD) measured at 600 nm (OD_600 nm_) of 1.0-2.0 at which point the culture was cooled to 18 °C. Protein expression was induced at 18 °C by adding 0.6 mM isopropyl β-D-1-thiogalactopyranoside (IPTG) (Biosynth), over expression was left to continue overnight (16 h). The level of over expressed construct was assessed by lithium dodecyl sulphate–polyacrylamide gel electrophoresis (LDS-PAGE) (Fig. 1). The prominent bands at around 65 kDa and 17 kDa correspond to the molecular weights of CaMKK2 and CaM, respectively.

**Fig. 2:**
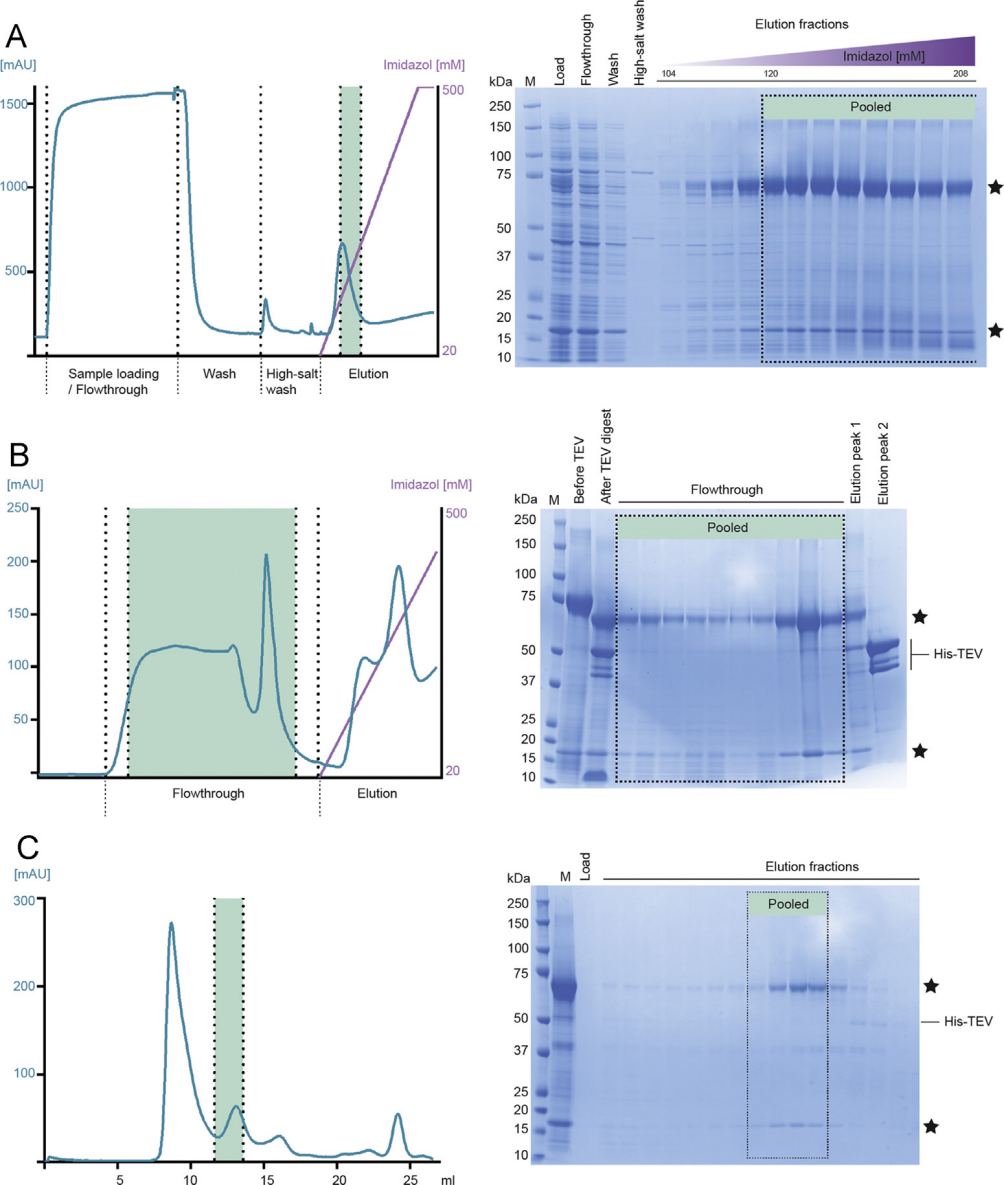
Purification of the CaMKK2:CaM protein complex. M=molecular weight marker. Bands corresponding to overexpressed CaMKK2 and CaM proteins are marked with an asterisk.

**Fig. 3:**
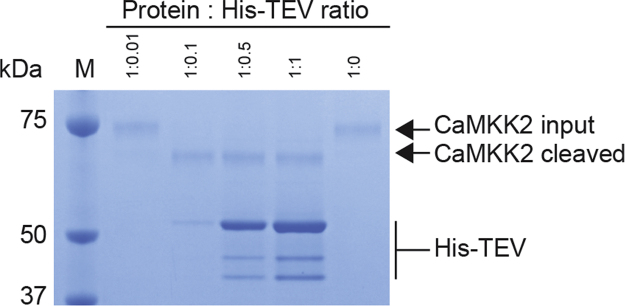
His-TEV test digest. M=molecular weight marker.

The multi-step purification of the CaMKK2:CaM complex was achieved over two days. All purification steps were carried out at 4 °C. The cells were harvested by centrifugation at 20,000*g* for 20 min and pellets were resuspended in lysis buffer (30 mM Hepes pH 7.2, 120 mM NaCl, 6 mM CaCl_2_, 20 mM imidazole, 8 mM beta-mercaptoethanol, 1 mM phenylmethylsulfonyl fluoride (PMSF; Nigu Chemie GmbH, Germany), 1 U/ml DNAse). The cells were lysed using a bead beater (Hamilton Beach, 908™; 0.1 mM glass beads) for 3×1 min and cellular debris was removed by centrifugation at 100,000*g* for 25 min. The His-tagged recombinant CaMKK2 along with un-tagged CaM was captured on a Ni-NTA Chelating HP column (GE Healthcare) in loading buffer (30 mM Hepes pH 7.2, 120 mM NaCl, 6 mM CaCl_2_, 20 mM imidazole, 8 mM beta-mercaptoethanol, 1 mM PMSF), followed by extensive washing with more than 20 column volumes of loading buffer and a high salt wash step (30 mM Hepes pH 7.2, 1 M NaCl, 6 mM CaCl_2_, 20 mM imidazole, 8 mM beta-mercaptoethanol, 1 mM PMSF) to remove the high amount of contaminants present. The chromatogram (Fig. 2A left) shows the absorbance profile (blue) and the imidazole gradient (purple) from 20 to 500 mM which was applied over 100 ml to elute the protein. Load, flow-through, wash steps, and eluate fractions were collected, separated by LDS-PAGE using precast NuPAGE 4–12 % Bis-Tris gels (LifeTechnologies) and subsequently stained with Coomassie (50 % EtOH, 10 % AcOH, 0.1 % Coomassie Brilliant Blue) to determine purity. The corresponding LDS-PAGE gel (Fig. 2A right) shows equal volumes of the load, flow-through, wash and high-salt wash material. Following, there are shown twelve elution steps, corresponding to 104–208 mM imidazole. Fractions corresponding to 120–228 mM imidazole (green highlighted) were pooled and subsequently incubated with purified His-TEV protease [3] to cleave off the N-terminal His-tag. To identify the optimal ratio, we initially digested Ni-NTA purified protein complex of CaMKK2:CaM with His-TEV protease in different ratios. A ratio of 1 : 0.1 (protein : His-TEV) gave sufficient cleavage, indicated by a clear shift in size of His_6_-tagged CaMKK2 to TEV-digested CaMKK2 (Fig. 3). This ratio was used for subsequent digests. Protein samples were further dialysed overnight in 30 mM Hepes pH 7.2, 120 mM NaCl, 6 mM CaCl_2_, 8 mM beta-mercaptoethanol. Un-cleaved CaMKK2 was removed by applying the digest a Ni-NTA column equilibrated in loading buffer, by collecting the flow through un-cleaved CaMKK2:CaM would stay on the column while leaving the cleaved product to flow through (Fig. 2B). We estimate that > 90 % of the His-tag was removed by this procedure (Fig. 2B, elution peak 1 and 2). The corresponding LDS-PAGE gel shows equal volumes of the pooled protein sample before and after incubation with TEV protease. Following, there are shown ten flow through steps, corresponding to the un-tagged protein sample. The imidazole elution peaks correspond to the His-TEV protease. Flow through fractions (green highlighted) were pooled and concentrated using an Amicon® Stirred Cells concentrator with a cut-off membrane of 30 kDa. The purification was finalized with a fine polishing step performed on size-exclusion chromatography (SEC), carried out in 30 mM Hepes pH 7.2, 120 mM NaCl, 6 mM CaCl_2_, 5 mM dithiothreitol (DTT) by injecting the protein in 0.5 ml increments into a Superdex 200 10/300 GL column (GE Healthcare) using a 10 ml superloop (GE healthcare). The spectral trace from the column (Fig. 2C, blue) revealed a particularly prominent peak in the void volume which did not correspond to visible protein on the corresponding LDS-PAGE gel (Fig. 2C) on which all main peaks between 7 and 25 ml were validated. The lower molecular band at ~50 kDa that elutes after CaMKK2:CaM we expect is remaining TEV protease, separated at this point. Importantly, the CaMKK2:CaM complex co-eluted visibly on the gel in the highlighted fractions (green) corresponding to a molecular weight of 78 kDa and indicating a 1 : 1 complex. These fractions were pooled and concentrated rated in Vivaspin 20 concentrators (Satorius stedim biotech, 10 kDa MWCO) for further validation and characterisation studies. Protein concentration was determined with the Nanodrop2000 (ThermoFisher scientific) using calculated extinction coefficients (Expasy/ProtParam).

### 2.3 Expression and purification of CaMKK2, CaMKK2(165-501):CaM complex, and CaMKK2 F267G

**Fig. 4:**
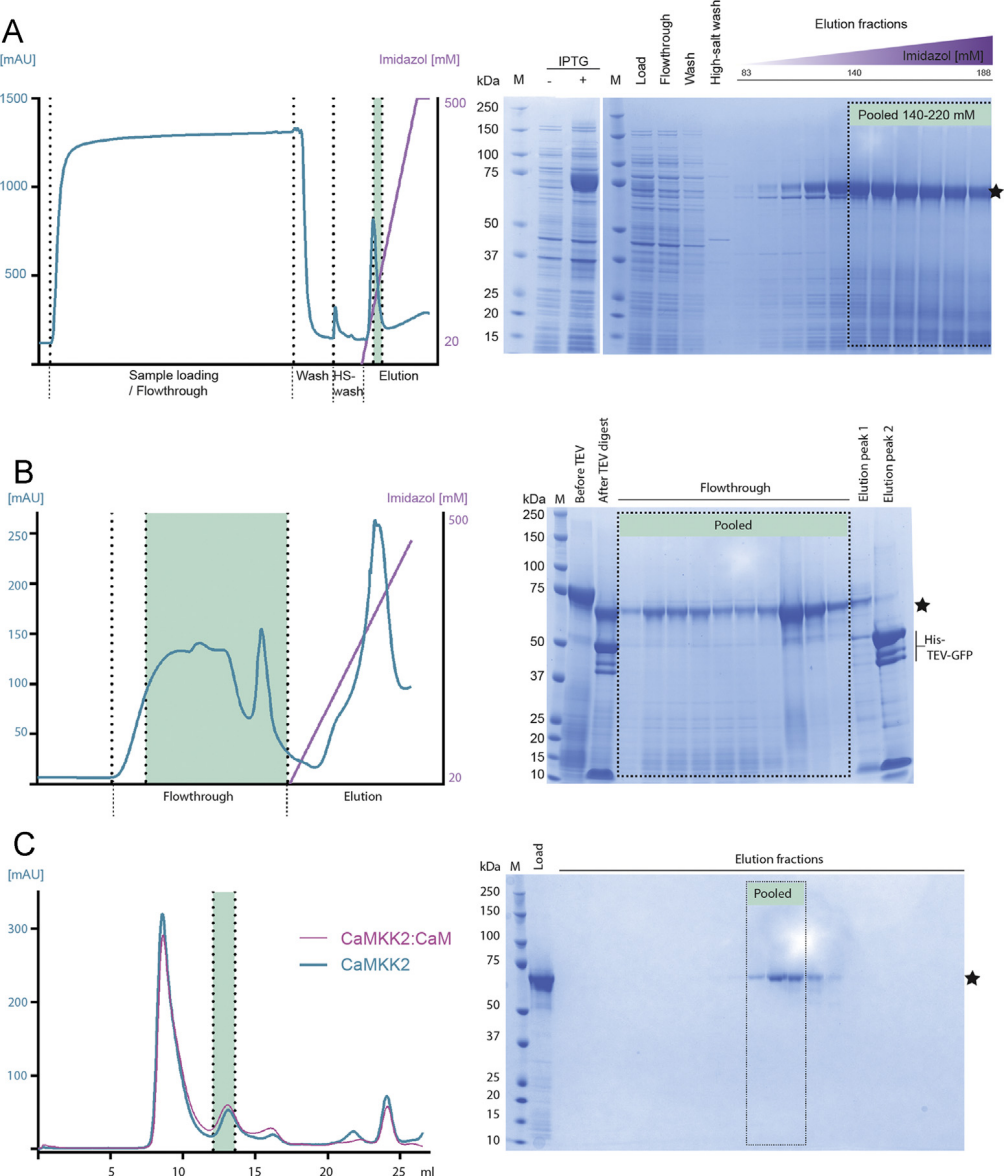
Purification of apo-CaMKK2. M=molecular weight marker. Bands corresponding to overexpressed CaMKK2 protein are marked with an asterisk.

An equivalent expression and purification was performed for CaMKK2 (without CaM) (Fig. 4). Notably, the spectral trace from the column (Fig. 4C, blue) revealed an almost identical profile compared to CaMKK2:CaM (purple) which may reflect that CaMKK2 in solution is more flexible and thus mimic protein of larger hydrodynamic volume, while the CaMKK2:CaM complex form a more compact spherical hetero dimer. Again, the prominent peak in the void volume did not correspond to visible protein on the corresponding LDS-PAGE gel on which all main peaks between 8 and 25 ml were validated. Importantly, CaMKK2 eluted visibly on the gel in the highlighted fractions (green) corresponding to a molecular weight of 65 kDa. Thus making further separation of CaMKK2 and CaMKK2:CaM complex inefficient via SEC, the single profiles do however show that the proteins seem monodisperse in solution. The unbound CaM was untagged and would have eluted earlier in the purification process.

## Funding sources

LG and IGM are supported in Oslo by funding from the Norwegian Research Council, Helse Sør-Øst and the University of Oslo through the Centre for Molecular Medicine Norway (NCMM), which is a part of the Nordic EMBL (European Molecular Biology Laboratory) partnership. IGM holds a visiting scientist position with Cancer Research UK through the Cambridge Research Institute and a Senior Honorary Visiting Research Fellowship with Cambridge University through the Department of Oncology. IGM is supported in Belfast by the Belfast-Manchester Movember Centre of Excellence (CE013_2-004), funded in partnership with Prostate Cancer UK. JKJ was supported from the Danish Cancer Society (R56-A2997). PM was supported from the Norwegian Cancer society (project #4483570).

## Figures and Tables

**Table 1:** The primers include pET-30/Ek-LIC specific sequences (underlined) and CaMKK2 gene sequence construct overlab in bold, *Bam*HI and *Xho*I sites (*italic*). Sequence for the TEV site (grey) coding for ENLYFQG. All reverse primers include a stop codon in parentheses. Primers given for the CaMKK2 F267G mutation only describe the primers used in the quickchange kit.

**Cloning**		
		Sequence 5′–3′
		
CaMKK2 into pET-30/Ek-LIC	fwd	GACGACGACAAGATGGAGAATCTTTATTTTCAGGGC**TCATCATGTGTCTCTAGCCAG**
	rev	GAGGAGAAGCCCGG(TTA)**CAAGAGCACTTCCTCCTCCCC**
		
CaMKK2 into pET28a	fwd	GC*GGATCC**T*****CATCATGTGTCTCTAGCCAG**
	rev	GGCCTCGAG(TCA)**CAAGAGCACTTCCTCCTCCCC**
		
CaMKK2 F269G into pET28a	fwd	**TACATGGTG**GGC**GAACTGGTC**
	rev	**GACCAGTTC**GCC**CACCATGTA**
CaMKK2 165-501 into pET28a	fwd	GC*GGATCC***TATACCCTGAAGGATGAAATTG**
	rev	GGC*CTCGAG(*CTA)**CTCGAATGGGTTCCCAAAGG**

**Sequencing**		
		Sequence 5′–3′
		
CaMKK2-pET30-EK/LIC	fwd	TAATACGACTCACTATAGGG
	rev	CTAGTTATTGCTCAGCGG
		
CaMKK2-pET28a	fwd	TAATACGACTCACTATAGGG
	rev	GCTAGTTATTGCTCAGCGG
CaM -pET8a	fwd	CGCAAATGGGCGGTAGGCGTG
	rev	CAGGGTGCCGGTGATGCGGC

## References

[1] Gerner L., Munack S., Temmerman K., Lawrence-Daerner A.M., Besir H., Wilmanns M., Jensen J.K., Thiede B., Mills I.G., Morth J.P. (2016). Using the fluorescent properties of STO-609 as a tool to assist structure–function analyses of recombinant CaMKK2. Biochem. Biophys. Res. Commun..

[2] I. de Diego, J. Kuper, N. Bakalova, P. Kursula, M. Wilmanns, Molecular basis of the death-associated protein kinase-calcium/calmodulin regulator complex, 3 (2010) ra6 10.1126/scisignal.2000552 20103772

[3] Wu X., Wu D., Lu Z., Chen W., Hu X., Ding Y. (2009). A novel method for high-level production of TEV protease by superfolder GFP tag. J. Biomed. Biotechnol..

